# MicroRNA profiling in women with migraine: effects of CGRP-targeting treatment

**DOI:** 10.1186/s10194-024-01787-2

**Published:** 2024-05-16

**Authors:** Raffaele Ornello, Veronica Zelli, Chiara Compagnoni, Valeria Caponnetto, Eleonora De Matteis, Cindy Tiseo, Alessandra Tessitore, Simona Sacco

**Affiliations:** 1 Department of Biotechnological and Applied Clinical Sciences, University of L’Aquila, Via Vetoio 1 Coppito, 67100 L’Aquila, Italy; 2 Department of Life, Health and Environmental Sciences, University of L’Aquila, L’Aquila, Italy; 3 Department of Brain Sciences, Imperial College London, London, UK; 4 Department of Neurology and Stroke Unit of Avezzano-Sulmona, Azienda Sanitaria Locale Di Avezzano-Sulmona-L’Aquila, Avezzano (L’Aquila), Italy

**Keywords:** Migraine prevention, Epigenetics, MicroRNA, Monoclonal antibodies, Erenumab, Biomarkers

## Abstract

**Background:**

Migraine lacks biomarkers that can trace the biological pathways of the disease and predict the effectiveness of treatments. Monoclonal antibodies targeting calcitonin gene-related peptide pathway – including erenumab – offer the opportunity of investigating potential migraine biomarkers due to their specific mechanism of action in preventing both episodic (EM) and chronic (CM) migraine. Our study aims at evaluating the expression levels of circulating microRNAs (miRNAs) according to migraine type, before and after treatment with erenumab and based on treatment response, in order to identify miRNAs with potential role as epigenetic biomarkers.

**Methods:**

The study included women aged 25–50 years with EM or CM treated with erenumab according to clinical indications. MiRNAs expression levels were assessed before (baseline) and after a 16-week treatment with erenumab, 140 mg every four weeks (post-treatment). An extensive miRNAs profiling was performed by qRT-PCR in small, pooled groups of ≤ 8 women each, classified according to migraine frequency (EM and CM) and the degree of response to erenumab. The expression levels of selected miRNAs were also validated using single miRNA assays in each woman with EM and CM.

**Results:**

During the study, 36 women with migraine (19 with EM and 17 with CM) out of 40 who were initially screened, performed the assessment of miRNA expression at baseline and post-treatment, Erenumab treatment significantly improved migraine burden in both EM and CM. MiRNA profiling revealed differential expression levels of a wide set of miRNAs (*hsa-let-7d-3p, hsa-miR-106b-3p, hsa-miR-122-5p, hsa-miR-143-3p, hsa-miR-144-3p, hsa-miR-16-5p, hsa-miR-181a-5p, hsa-miR-221-3p, hsa-miR-25-3p, hsa-miR-29b-2-5p, hsa-miR-326, miR-363-3p, hsa-miR-424-5p, hsa-miR-485-3p, hsa-miR-532-5p, hsa-miR-543, hsa-miR-629-5p, hsa-miR-660-5p, hsa-miR-92a-3p)* depending on treatment response. Among them, single miRNA assays confirmed the progressive decrease of *hsa-miR-143-3p* expression levels in relation to increasing response to erenumab in women with EM (7 with low, 6 with medium, and 6 with high response; *p* = 0.02). Additionally, single assays showed higher *hsa-miR-34a-5p* and *hsa-miR-382-5p* expression levels at baseline in women with CM compared with those with EM (*p* = 0.0002 and *p* = 0.0007, respectively), as well as their expression level decrease in women with CM from baseline to follow-up (*p* = 0.04 and *p* = 0.02, respectively).

**Conclusions:**

Our study suggests that targeting the CGRP pathway in migraine changes the expression levels of certain miRNAs. These miRNA levels are linked to the levels of response to CGRP receptor blockage. Future research challenges include assigning specific functions to the modulated miRNAs to unravel pathways modulated by the disease and the treatment.

**Trial registration:**

The study was registered in clinicaltrials.gov with code NCT04659226 and in the Novartis database with code CAMG334AIT05T.

**Supplementary Information:**

The online version contains supplementary material available at 10.1186/s10194-024-01787-2.

## Background

Migraine, one of the most common neurological disorders [1], imposes substantial disability burden on society [2]. The International Classification of Headache Disorders, 3rd edition (ICHD-3), distinguishes episodic (EM) and chronic migraine (CM) [3], with CM bearing the greatest disease burden [4].

Treatments targeting the calcitonin gene-related peptide (CGRP) pathway such as monoclonal antibodies and gepants are the first drugs designed to prevent migraine by acting on a specific mechanism – CGRP release from the trigeminal ganglion [5]. Robust evidence from randomized controlled trials and real-world evidence supports the safety and efficacy of treatments targeting the CGRP pathway for both EM and CM prevention, endorsed by global guidelines [6–9]. Although there are various preventive treatments for migraine that might act on several mechanisms of the disorder, the specificity of agents targeting the CGRP makes them well-suited to probe the neurological and physiological changes associated with effective migraine prevention.

To date, the absence of established biomarkers hinders a precise classification and treatment of migraine due to the disorder's intricate biopsychosocial nature [10, 11]. Genome-wide association studies (GWAS) linked certain genes to migraine [12], yet their ability to predict migraine severity, frequency or other clinical characteristics remains limited. Epigenetic markers, particularly microRNAs (miRNAs), are emerging as promising candidates for migraine characteristics, prognosis, and response to treatments [13, 14]. miRNAs might also be used as treatment targets, even if they are not currently considered to design novel migraine treatments. Notably, some studies assessed the change in the expression of specific miRNAs in patients with migraine after acute [15] or preventive [16] migraine treatment. The availability of drugs specifically designed to prevent migraine offers the opportunity of investigating the epigenetic modulation of migraine activity by linking it to pathogenetic mechanisms.

This study aims at providing new insights into miRNA expression profiles in women with EM and CM undergoing a migraine-specific treatment. A broad assessment of miRNA changes offers a potentially novel approach to better understand migraine pathogenesis, distinguish EM from CM and elucidate changes associated with effective prevention as well as highlight miRNAs with potential role as biomarkers of treatment response.

## Methods

### Study design

We conducted an interventional, non-randomized study in which miRNA expression levels were assessed before (baseline) and after (post-treatment) treatment with subcutaneous injections of erenumab 140 mg every four weeks. The 140 mg dose was chosen over the 70 mg dose to limit heterogeneity of treatment and maximize efficacy, as the 140 mg dose showed advantages over the 70 mg dose [17, 18].

### Study population

The study enrolled adult women seeking follow-up visits at the Headache Center of ASL Avezzano-Sulmona-L’Aquila. Women with EM and CM were consecutively recruited in parallel to ensure balanced subgroup sizes.

Inclusion criteria were:

- Female sex;
- Age 25–50 years;
- Documented EM or CM based on International Classification of Headache Disorders (ICHD-3) criteria [3];
- Minimum one-year migraine history;
- Clinical indication to erenumab treatment per Summary of Product Characteristics [19] and Italian reimbursement criteria [20];
- Consistent headache tracking using paper or electronic diary for ≥ 3 months pre-baseline;
- Written informed consent.

Exclusion criteria were:

- Non-migraine headache disorders;
- Prior erenumab/CGRP-mAb exposure;
- Pregnancy/nursing;
- Body mass index < 18 or > 30 kg/m^2^;
- Heavy smoking (> 20 cigarettes daily);
- History of heart ischemia or procedures;
- Illicit drug abuse;
- Severe psychiatric disorders;
- Active infections/inflammation;
- Known erenumab-related adverse reactions per Summary of Product Characteristics [19];
- Any major comorbidities or serious medical condition at the discretion of the treating physician.

Women with medication overuse headache were eligible if meeting ICHD-3 criteria for migraine [3]. To establish the correct diagnosis and treatment for women with medication overuse headache, we ensured that the onset of migraine preceded the onset of medication overuse. Thus, we could consider medication overuse as a complication of migraine [21]. To limit confounding, participants on concomitant medications could join the study only if dosage and schedule of the medication remained stable. Participants were advised to maintain diet, sleep, and physical activity levels to prevent miRNA changes due to parameter shifts.

Women of childbearing potential were encouraged to use contraception (abstinence, hormonal, barrier, or sterilization) due to the untested pregnancy effects of erenumab.

### Visit schedule and procedures

The study utilized subcutaneous administrations of erenumab 140 mg every four weeks as the treatment approach. Erenumab prescription and administration followed an open-label design, adhering to Italian reimbursement criteria [20]. In detail, women were eligible for erenumab reimbursement if they experienced ≥ 8 monthly days of debilitating headaches and held a Migraine Impact and Disability Assessment Scale (MIDAS) score of ≥ 11. Furthermore, participants needed to demonstrate inadequate responses (due to inefficacy, intolerance, or contraindication) to ≥ 3 treatment classes, which included beta-blockers, anticonvulsants, tricyclic antidepressants, and onabotulinumtoxinA (for chronic migraine), following ≥ 6 weeks of treatment at appropriate doses.

Throughout the study duration, participants retained the freedom to utilize their standard abortive medications for managing migraine episodes. The initiation of new abortive medications during the study period was discouraged, aiming to maintain consistency and avert potential changes that could influence miRNA expression.

After signing the informed consent, participants were assigned a code and underwent a baseline visit in which we collected age, race/ethnicity, vital signs – blood pressure, heart rate, height, and weight – and relevant details on medical history. Headache history was collected by reviewing the women’s headache diaries during the four weeks prior to baseline. Compliance with headache diaries is encouraged as common clinical practice in the headache center and used as an essential instrument to monitor response to any prescribed treatment. Parameters assessed for the present study included headache days, migraine days, days of consumption of acute migraine medications, and doses of consumption of those medication. We also recorded patient-reported outcomes (PROs) including the MIDAS, Headache Impact Test (HIT-6), Beck Depression Inventory (BDI), Allodynia Symptom Checklist-12 (ASC-12), and Pittsburgh Sleep Quality Inventory (PSQI). During the baseline visit, women also underwent blood sampling for baseline miRNA expression analysis.

The baseline visit coincided with clinical decision to prescribe erenumab. As erenumab is designed for self-injection, up to one week was given to patients to get erenumab pre-filled syringes and start treatment. Participants who signed informed consent but failed to start treatment for any reason were excluded from analyses.

At the baseline visit, participants were encouraged to comply with the headache diary. Headache and migraine frequency, analgesic and triptan consumption were monitored and reported for 12 weeks – corresponding to three erenumab administrations – after treatment start. This time was considered as the minimum time to allow stable CGRP receptor inhibition. The original study protocol planned a follow-up visit after this 12-week period. However, according to the Italian reimbursement procedures [20] which were issued after the release of the original study protocol, patients must be clinically reassessed after 16 weeks of treatment with erenumab. Therefore, for practical reasons, we decided to reassess patients with a post-treatment visit after 16 weeks of treatment. The post-treatment visit included a recording of headache and migraine frequency and analgesic consumption during the first 12 weeks of treatment, vital signs, PROs, and a new blood sampling for miRNA expression evaluation. Adverse events (AEs) and serious adverse events (SAEs) to erenumab were also collected and reported according to common clinical practice.

Participants not attending the post-treatment visit were excluded from data analyses.

### Sample collection

Whole blood samples of screened women were collected in EDTA-containing tubes by the clinical investigators directly at the headache center at both the screening and the follow-up visit. Baseline and follow-up samples from the same women were paired.

Plasma samples were obtained by double centrifugation (10 min at 1,000 × g and 10 min at 3,000 × g, using a + 4 °C refrigerated centrifuge) within an hour from blood collection at the ‘Tecniche di Medicina di Laboratorio’ Laboratory at the Department of Biotechnological and Applied Clinical Sciences, University of L’Aquila, located at a 10-min walking distance from the headache center. Plasma samples were long-term stored at -80 °C. To comply with legal requirements for the storage of genetic material, the laboratory personnel had access to the patients’ identification codes but not to their clinical data.

### RNA extraction and miRNA profiling by microfluidic cards in pooled samples

To perform miRNA testing, total RNA was extracted from 500ul of women’s plasma using the mirVana PARIS RNA Purification Kit (Thermo Fisher, Waltham, MA, USA), following the manufacturer’s protocol. RNA quantity was assessed using Qubit Fluorometer (Invitrogen, Thermo Fisher). Afterwards, RNAs were pooled together in groups of ≤ 8 patients according to migraine type (EM or CM) and response to erenumab (see Statistical analysis for reference) and 2 ng of total RNA were retrotranscribed using TaqMan Advanced miRNA cDNA Synthesis Kit (Applied Biosystem) according to the manufacturer’s protocol. This step consists of four phases which include the poly(A) tailing reaction, the adaptor ligation reaction, the reverse transcription (RT) reaction and the miR-Amp reaction.

MiRNA profiling was performed by qRT-PCR, by using microfluidic cards (TaqMan Advanced miRNA Human Serum/Plasma Cards, Thermo Fisher), which allow to evaluate the expression profile of 189 unique miRNAs and two exogenous controls (*ath-miR159a and cel-miR-39-3p*) with high sensitivity and specificity, starting from a very low amount of total RNA input. Notably, *ath-miR159a* was added during RNA extraction and used to normalize miRNA expression data, while *cel-miR-39-3p* was added during cDNA synthesis and used to monitor the efficiency of the reaction and qRT-PCR. Details of the protocol used for cDNA synthesis and qRT-PCR analysis with TaqMan array cards can be found at https://assets.thermofisher.com/TFS-Assets/LSG/manuals/MAN0016123_TaqManAdvmiRNAArrayCards_QR.pdf.

Samples were analyzed on a ViiA7 instrument (Applied Biosystems, Thermo Fisher) allowing the profiling of up to 188 unique miRNAs, representing the most widely characterized circulating miRNAs. The complete list of analyzed miRNAs is provided in Supplementary Table 1. Two card replicates, allowing the analysis of each target in quadruplicate, were considered for each pooled sample.

### Validation analysis by single miRNA assay on individual samples

MiRNAs whose levels changed according to different subtypes of migraine (EM or CM), different timepoints (baseline or post-treatment), or degree of response to erenumab, as identified by miRNA profiling, were further validated by single assay in individual samples (TaqMan Advanced miRNA Assay, Life Technologies). In addition to the most interesting miRNAs that showed changes in profiling analysis, we assessed the expression levels of two miRNAs (*hsa-miR-34a-5p* and *hsa-miR-382-5p*), previously described as dysregulated in similar case series of patients with migraine [16, 22]. Starting from 2 ng of total RNA input, reverse transcription was performed using TaqMan Advanced miRNA cDNA Synthesis Kit (Applied Biosystem) following manufacturer’s protocol and qRT-PCR was carried out on a ViiA7 instrument (Life Technologies). Details of the protocols used for cDNA synthesis and qRT-PCR analysis with single miRNA assays can be found at https://assets.thermofisher.com/TFS-Assets/LSG/manuals/100027898_TaqManAdv_miRNA_Assays_QR.pdf.

### Study outcomes

The study had three objectives, all related to the identification of differentially expressed miRNAs, by comparing:

1. women with EM and with CM, at baseline and post-treatment;
2. miRNA levels from baseline to post-treatment in each group of women – those with EM and those with CM;
3. miRNA levels, based upon the extent of response to erenumab, in women with EM and with CM.

All those objectives were evaluated in both card assays and single assays on selected miRNAs. Overall, the expected outcome was the identification of miRNAs to be putatively considered as biomarkers.

### Data analysis

Continuous data were summarized as mean with standard deviation (SD), while categorical data were presented as absolute and relative frequencies (numbers and proportions). To ensure comparability with other real-world evidence for erenumab, we documented reductions in mean headache days, migraine days, days, and doses of acute medication consumption from baseline to weeks 1–4, 5–8, and 9–12 of follow-up with paired t-tests. Additionally, paired t-tests were used to report changes in PROs from baseline to follow-up.

Data analysis of miRNA cards was performed using QuantStudio Software v 1.3 and Expression Suite software v 1.3 (Thermo Fisher). MiRNA expression levels (RQ, relative quantification) were assessed by comparative assay (2^−ΔΔCt^) and data were normalized using ath-miR159a exogenous control and global normalization method. A manual check focused on PCR amplification plots profiles was also performed and only miRNAs with good amplification curves were retained. Differentially expressed miRNAs between different groups were filtered based on RQ < 0.5 for down-regulated miRNAs and RQ > 2 for up-regulated miRNAs, and a *p*-value < 0.05.

Regarding single assays, ath-miR159a was considered as exogenous control and, to assess the strength of data, global normalization method was also used. Each sample was run in triplicate and relative miRNA expression levels were determined by the ΔCt method and expressed as 2^–ΔCt^. The non-parametric Mann–Whitney-Wilcoxon or Kruskal–Wallis test was used to compare relative expression levels of each miRNA in the different study groups. Receiver operating characteristic (ROC) curves were used to estimate the diagnostic value of selected miRNAs by using the *CombiROC* package. Where more than one miRNA was associated to treatment response, ROC curves were built on the combination of those miRNAs.

The analyses were performed with the R software (www.r-project.org) and a *p*-value < 0.05 was considered statistically significant.

Following the objectives of the present study, group analyses included differences in miRNAs levels in women with EM and CM, changes in miRNAs levels from baseline to follow-up, and stratification of women with EM and CM based on erenumab response tertiles (low, medium, high), defined by percent change in monthly migraine days from baseline to weeks 9–12 of follow-up. The latter classification was carried out to identify miRNAs with potential role as predictive biomarkers of treatment response (baseline analysis) and miRNAs modulated by or involved in treatment response-induced mechanisms (post-treatment analysis).

For sample size determination, a cohort of 40 evaluable patients was calculated to yield a 95% confidence interval for mean changes with a ± 0.248 width, considering a standard deviation of 0.8 [22]. To ensure sufficient power for subgroup analyses of women with EM and CM, the 95% confidence interval width was adjusted to ± 0.351, corresponding to a sample size of 20 patients each for EM and CM subgroups.

### Ethical aspects

The study was approved by the Ethics Committee for the districts of L’Aquila and Teramo with protocol number 417/20, registered in clinicaltrials.gov with the reference code NCT04659226 and in the Novartis databases with code CAMG334AIT05T. All patients signed informed consent for inclusion in the present study.

## Results

**Fig. 1:**
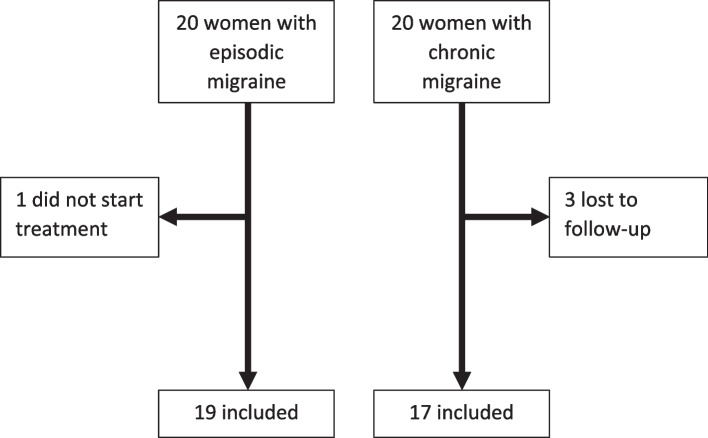
Flowchart of womens’ inclusion

Over the study period (July 2021 – December 2022), 40 women with migraine (20 with EM and 20 with CM) were initially recruited. One woman with EM did not start treatment with erenumab, while three women with CM were lost to follow-up; therefore, we ended up with 36 women, 19 with EM and 17 with CM (Fig. 1).

### Baseline characteristics

Table 1 shows the characteristics of women included in the study. No statistically significant differences emerged between women with EM and CM regarding baseline characteristics.

**Table 1:** Baseline characteristics of women included in the present study

	**Overall (*n* = 36)**	**EM (*n* = 19)**	**CM (*n* = 17)**	***p* value (EM vs CM)**
Age (years), mean ± SD	39.6 ± 7.9	36.9 ± 8.0	42.5 ± 6.9	0.033
Years of migraine history, mean ± SD	23.3 ± 7.9	23.4 ± 10.1	23.2 ± 12.1	0.962
Aura, n (%)	9 (25.0)	2 (10.5)	7 (41.2)	0.083
Medication overuse, n (%)	12 (33.3)	-	12 (70.6)	-
Failures^a^, n (%)				
Antidepressants	35 (97.2)	19 (100.0)	16 (94.1)	0.713
Anticonvulsants	36 (100.0)	19 (100.0)	17 (100.0)	0.965
Beta-blockers	35 (97.2)	19 (100.0)	16 (94.1)	0.499
Calcium channel blockers	13 (36.1)	10 (52.6)	3 (17.6)	0.085
ACE inhibitors /sartans	-	-	-	-
OnabotulinumtoxinA	3 (8.3)	-**	3 (17.6)	-**
Other	6 (16.7)	5 (26.3)	1 (5.9)	0.232
Vital signs				
BMI, mean ± SD	22.2 ± 4.0	21.7 ± 3.7	22.9 ± 4.4	0.385
Systolic blood pressure, mean ± SD	112.6 ± 17.5	114.8 ± 20.7	110.0 ± 13.1	0.403
Diastolic blood pressure, mean ± SD	73.2 ± 12.4	72.6 ± 13.5	73.9 ± 11.3	0.755
Heart rate, mean ± SD	73.7 ± 9.9	71.5 ± 9.6	76.1 ± 10.0	0.176

^a^due to either ineffectiveness, intolerance, or contraindication

^**^OnabotulinumtoxinA is authorized for use only in women with CM

### Efficacy and safety data

**Fig. 2:**
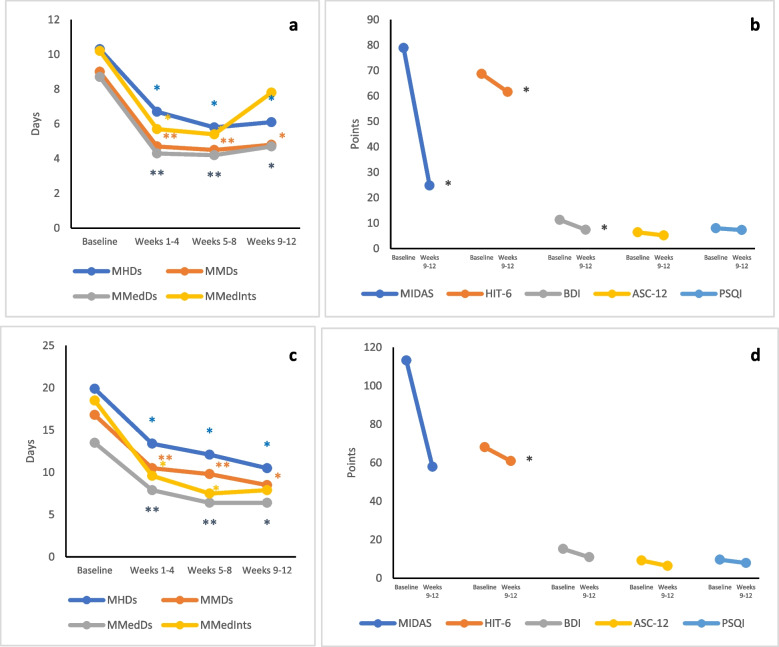
Mean change in efficacy outcomes of erenumab in women with episodic migraine (**a**, **b**) and chronic migraine (**c**, **d**) in the present study. *p < 0.05 **p < 0.001. MMDs indicates monthly migraine days; MHDs, monthly headache days; MMedDs, monthly medication days; MMedInts, monthly medication intakes; MIDAS, Migraine Impact and Disability Assessment Scale; HIT-6, Headache Impact Test-6; BDI, Beck Depression Inventory; ASC-12, Allodynia Symptom Checklist-12; PSQI, Pittsburgh Sleep Quality Index

Effectiveness data of erenumab treatment in women with EM and with CM are reported in Supplementary Table 2 and in Fig. 2. Overall, erenumab treatment significantly improved migraine burden in both groups. Four participants with EM had adverse events, one which was considered serious; however, none of them stopped erenumab treatment. Nine participants with CM had adverse events, one of which was considered serious; however, none of them stopped the treatment.

### miRNA profiling by microfluidic cards in pooled samples

miRNA profiling, performed in pooled group of samples, revealed no significant difference at baseline or post-treatment in expression levels of the tested miRNAs comparing the whole group of women with EM with the group of women with CM (first objective of the study). No difference was found from baseline to post-treatment, either in participants with EM or in those with CM (second objective of the study).

**Fig. 3:**
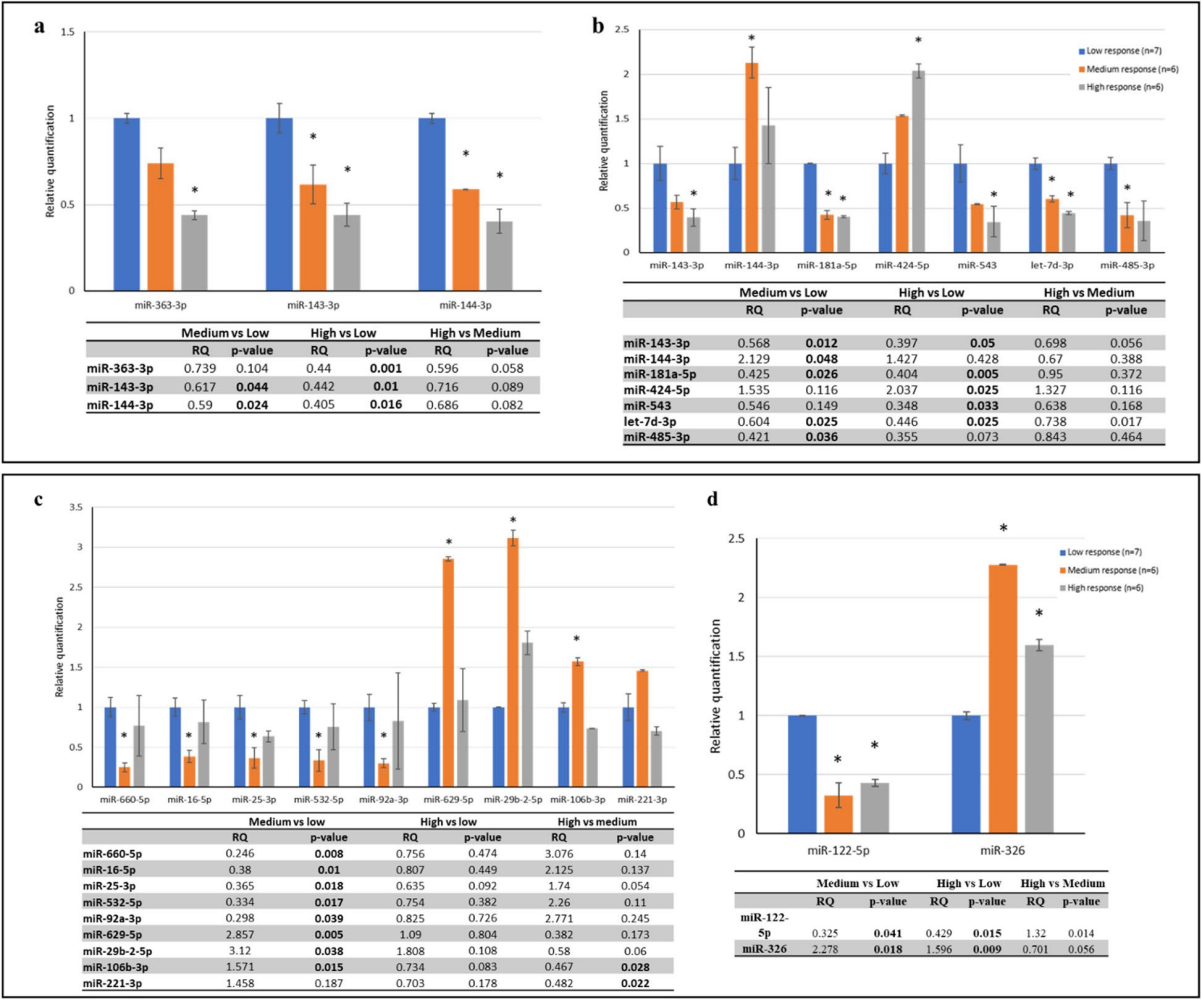
Change in levels of microRNAs at baseline (**a**) and post-treatment (**b**) in women with episodic migraine according to erenumab response. Change in levels of microRNAs at baseline (**c**) and post-treatment (**d**) in women with chronic migraine according to erenumab response. *p < 0.05; significant p-values are in bold. RQ indicates relative quantification

In reference to the third objective of the study, Table 2 reports the cut points for tertiles of response to erenumab. In women with EM, we found that the expression levels of *hsa-miR-363-3p, hsa-miR-143-3p,* and *hsa-miR-144-3p* at baseline decreased progressively with increasing response to erenumab (Fig. 3a). In women with CM, the expression levels of five miRNAs (*hsa-miR-660-5p, hsa-miR-16-5p, hsa-miR-25-3p, hsa-miR-532-5p* and *hsa-miR-92a-3p*) at baseline were lower in the subgroup with medium response compared with low or high response to erenumab, while the expression levels of three miRNAs (*hsa-miR-629-5p, hsa-miR-29b-2-5p* and *hsa-miR-106b-3p*) at baseline were higher in the subgroup with medium response compared with low or high response to erenumab (Fig. 3c).

**Table 2:** Categories of response to erenumab in patients with episodic and chronic migraine. Proportions indicate change from baseline to post-treatment in monthly migraine days

	n	Min	Max
Episodic migraine (*n* = 19)			
Low response	7	+ 42%	-40%
Medium response	6	-47%	-71%
High response	6	-88%	-100%
Chronic migraine (*n* = 17)			
Low response	5	+ 3%	-26%
Medium response	6	-38%	-68%
High response	6	-74%	-100%

In women with EM, the expression levels of five miRNAs (*hsa-miR-143-3p, hsa-miR-181a-5p, hsa-miR-543, hsa-let-7d-3p,* and *hsa-miR-485*) post-treatment decreased, while those of two miRNAs (*hsa-miR-144-3p* and *hsa-miR424-5p*) increased progressively with increasing response to erenumab (Fig. 3b). In women with CM, the expression levels of *hsa-miR-122-5p* at follow-up were lower in the subgroup with medium or high response compared with low response to erenumab, while the expression levels of *hsa-miR-326* at follow-up were higher in the subgroup with medium or high response compared with low response to erenumab (Fig. 3d).

### Validation analysis by single miRNA assay on individual samples

Single assay analysis was performed in individual samples on the eleven miRNAs showing, either at baseline or follow-up, different expression levels according to response to erenumab in the profiling analysis (*hsa-miR-363-3p, hsa-miR-143-3p, hsa-miR-144-3p, hsa-miR-181a-5p, hsa-miR-424-5p, hsa-miR-543, hsa-miR-485-3p, hsa-miR-25-3p, hsa-miR-29b-2-5p, hsa-miR-122-5p* and *hsa-miR-326*) and on *hsa-miR-34a-5p* and *hsa-miR-382-5p* which were reported as dysregulated in migraine by previous studies [16, 22];of note, the expression levels of these two miRNAs were not evaluated in card analysis due to the low quality of their amplification curves, as described in *Data analysis* section.

**Fig. 4:**
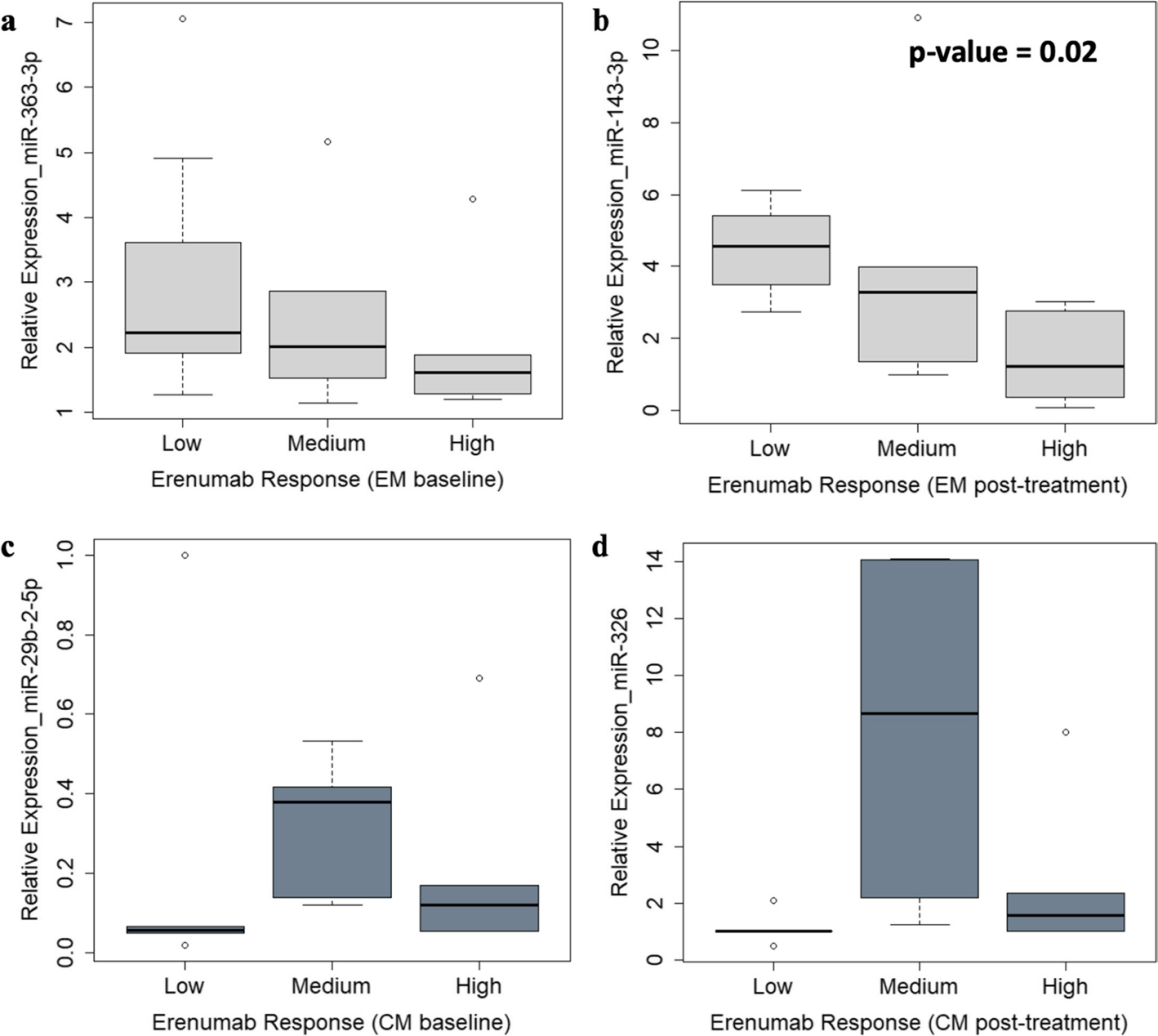
Boxplot showing miRNA expression levels in individual women with episodic or chronic migraine according to erenumab response, validated using single miRNA assays: miR-363-3p in women with episodic migraine at baseline (**a**), miR-143-3p in women with episodic migraine post-treatment (**b**), miR-29b-2-5p in women with chronic migraine at baseline (**c**) and miR-326 in women with chronic migraine post-treatment (**d**). Statistically significant p-values are reported

As shown in Fig. 4, we found significant expression level decrease of *hsa-miR-143-3p* at follow-up with increasing response to erenumab in women with EM (*p* = 0.02; Fig. 4b). Non-significant changes included a trend toward decreasing expression levels of *hsa-miR-363-3p* at baseline with increasing response to erenumab in the EM group (Fig. 4a), a trend toward higher expression levels of *hsa-miR-29b-2-5p* at baseline and of *hsa-miR-326* at follow-up in the medium response subgroup compared to low or high response in women with CM (Fig. 4c-d).

**Fig. 5:**
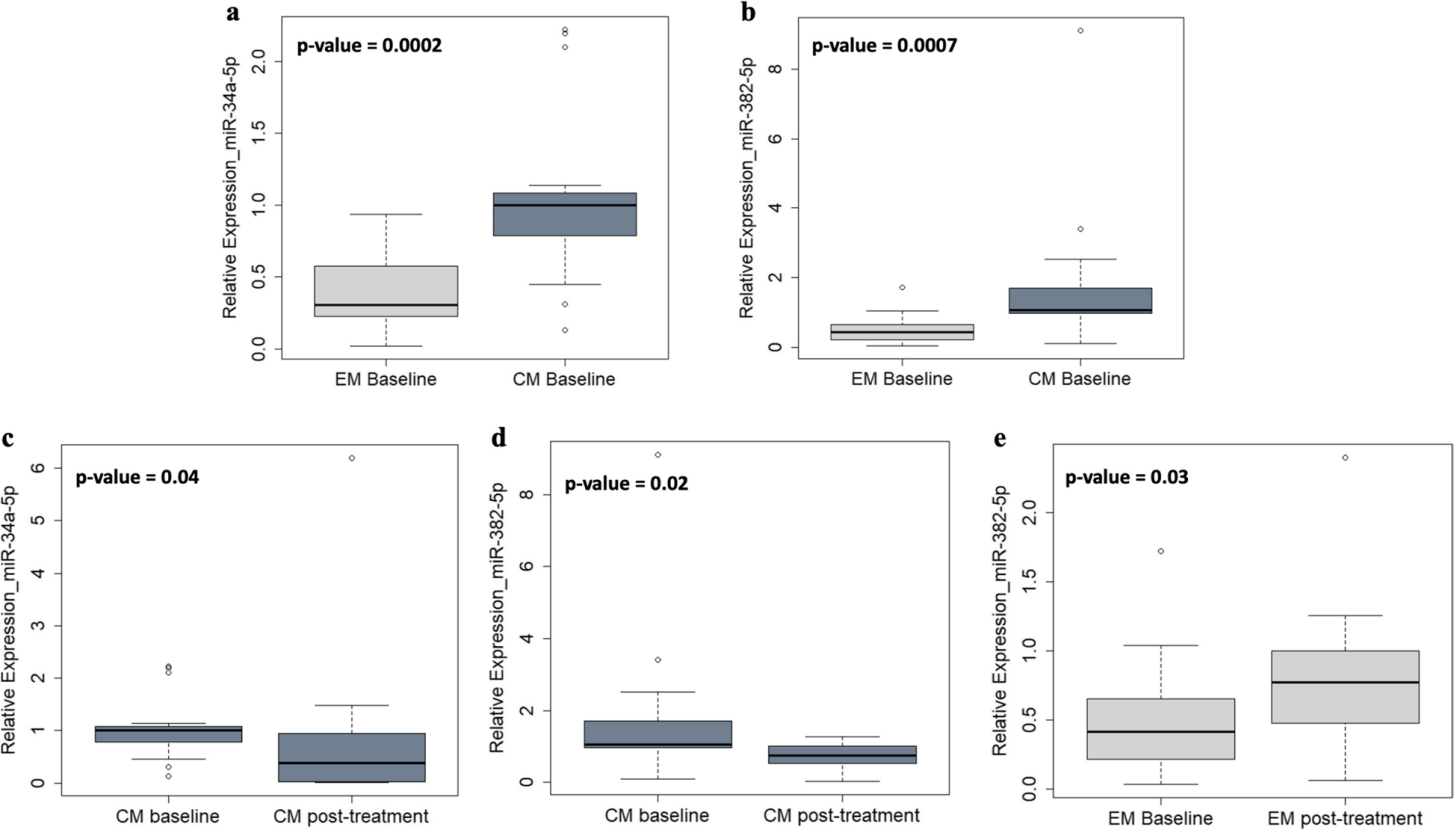
Boxplot showing miR-34a-5p and miR-382-5p expression levels in women with episodic or chronic migraine, validated using single miRNA assays. **a**,**b** miR-34a-5p and miR-382-5p expression levels in women with chronic migraine compared to those with episodic migraine. **c**,**d** miR-34a-5p and miR-382-5p expression levels in women with chronic migraine post-treatment. **e** MiR-382-5p expression levels in women with episodic migraine post-treatment. Only statistically significant p-values are reported

Considering *hsa-miR-34a-5p* and *hsa-miR-382-5p* [16, 22]*,* significantly higher expression levels of both at baseline were observed in women with CM compared to those with EM (*p* = 0.0002 and *p* = 0.0007 for *hsa-miR-34a-5p* and *hsa-miR-382-5p*, respectively) (Fig. 5a-b), as well as a down-regulation from baseline to post-treatment in women with CM (*p* = 0.04 and *p* = 0.02 for *hsa-miR-34a-5p* and *hsa-miR-382-5p*, respectively) (Fig. 5c-d). A significant increase in post-treatment *hsa-miR-382-5p* expression level compared to baseline was found in women with EM (*p* = 0.03) (Fig. 5e).

**Fig. 6:**
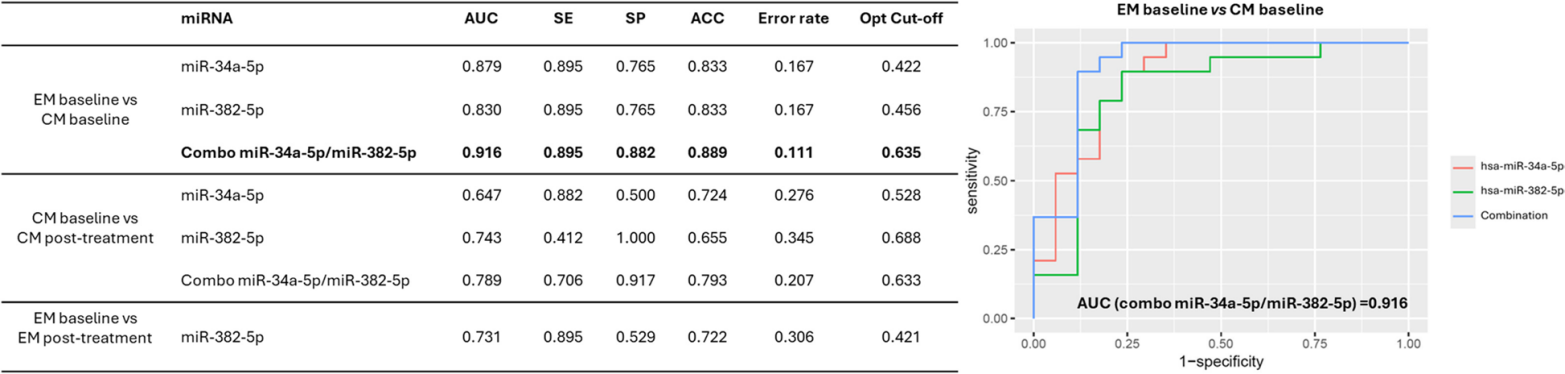
Results of the Receiver-Operating Characteristic curve analysis of miR-34a-5p and miR-382-5p to discriminate women with chronic migraine from those with episodic migraine and to assess the difference from baseline to post-treatment in each of the two groups. The most promising combination is highlighted in bold and shown in the plot. Abbreviations: Area Under the Curve (AUC), sensitivity (SE), specificity (SP), accuracy (ACC), optimal cutoff (Opt Cut-off)

ROC curve analysis was used to assess the diagnostic value of has-miR-34a-5p and has-miR-382-5p in discriminating women with CM from EM at baseline and in assessing the difference from baseline to post-treatment in each of those two groups (Fig. 6).

Interestingly, the combination of the two miRNAs showed very good diagnostic accuracy, higher than each single miRNA in discriminating women with CM from those with EM, with Area Under the Curve (AUC) of 0.916 and Accuracy (ACC) of 0.889. The optimal cut-off value was indicated at 0.635, which demonstrated a sensitivity of 0.895 and a specificity of 0.882. Similarly, the combination of the two miRNAs showed greater ability than the single miRNAs to discriminate women with CM at baseline compared to post-treatment, with AUC value of 0.789 and ACC of 0.793. The sensitivity and specificity of this combination were 0.895 and 0.882, respectively.

## Discussion

miRNAs are epigenetic factors that can be studied to improve the knowledge on migraine pathogenesis and patient classification [23]. To date, studies investigating the association between miRNAs and migraine are mainly based on the use of comprehensive miRNAs arrays, by comparing their expression levels to the general population, or are focused on a subset of miRNAs previously associated with the disorder. Prior well-conducted studies have identified miRNAs that are dysregulated in individuals with migraine [24–27]. Additionally, a previous study indicated that their expression levels can change with erenumab treatment [16]. Given the extensive number of miRNAs expressed in the body, each with a broad range of functions as epigenetic modulators, a broader assessment of miRNAs associated with migraine treatment response is crucial. This becomes particularly significant for recent migraine-specific preventive treatments, such as those targeting the CGRP pathway. Identifying these specific miRNAs could help finding migraine biomarkers and predicting treatment response. Additionally, miRNAs whose expression levels are modified by migraine or its treatments could also become potential therapeutic targets.

Here, we investigated circulating miRNA expression levels of a well-characterized series of women with EM and CM undergoing a CGRP-targeting treatment. It is noteworthy that our study population demonstrated similar efficacy of erenumab compared to previous real-world studies for both EM and CM [28–31]. Therefore, our population is representative of the general cohort of patients undergoing this treatment.

Regarding the first objective of our study – identifying differences in miRNA expression between women with EM and those with CM – our findings show increased expression levels of *hsa-miR-34a-5p* and *hsa-miR-382-5p* in participants with CM compared to EM, in line with previous literature [17]. We also showed the ability of these two miRNAs to discriminate with high accuracy women with EM with respect to those with CM, highlighting the potential diagnostic value of this two-miRNA signature. No novel miRNAs emerged as biomarkers able to distinguish CM from EM. However, we cannot exclude that more in-depth examination of the miRNome – e.g., by smallRNA-seq – might reveal additional miRNAs with potential role in classifying migraine. At the same time, it is worth noting that the differences between EM and CM in our study were not those of the general population. While in the general population CM is associated with a higher degree of disability and lower quality of life compared with EM [32], women with EM treated with erenumab, in accordance with reimbursement criteria, often have severe high-frequency EM, carrying a burden of disability akin to CM [33]. This similarity in burden could underlie the epigenetic likeness between EM and CM. Further exploration in a more differentiated population of patients might reveal epigenetic differences between EM and CM, as those differences might be implied in migraine chronification [34].

Regarding the second objective of our study – comparing post-treatment *vs* baseline miRNA expression levels – a decrease of *hsa-miR-34a-5p* and *hsa-miR-382-5p* was detected in women with CM after treatment as well as the good diagnostic accuracy of this two-miRNA signature..A significant *hsa-miR-382-5p* increase in women with EM by comparing post-treatment *vs* baseline was also observed. To date, very little is known about changes in the levels of circulating miRNAs in relation to CGRP-targeting treatments. A recent study reported decreased levels of *hsa-miR-34a-5p* and *hsa-miR-382-5p* in peripheral blood lymphocytes from patients with CM treated with erenumab [16]. Those same miRNAs were also down-regulated in our study, even if our tests were performed on plasma samples instead of peripheral blood cells and in women who did not undergo detoxification for medication overuse before starting the monoclonal antibody. Notably, we observed decreased expression levels of *hsa-miR-34a-5p* and *hsa-miR-382-5p* in women with CM post-treatment and an opposite trend for *hsa-miR-382-5p* in women with EM. Our study was the first to assess the effect of a CGRP-targeting treatment on epigenetic markers in women with EM, as the previous study only included individuals with CM [16]. *Hsa-miR-34a-5p* and *hsa-miR-382-5p* might become interesting biomarkers of migraine preventive treatment; however, it is worth noting that those miRNAs were not shown, to date, to interact with CGRP pathways [35].

Regarding the third objective of our study – conducting subgroup analyses based on treatment response –, miRNA profiling revealed significant differences in miRNA expression levels according to the degree of response (low, medium, high) which are specifically related to the type of migraine (EM or CM). Among these, *hsa-miR-143-3p* was modulated post-treatment in women with EM according to treatment response, and emerged as the most robust biomarker, being also confirmed by single assay analysis. Further investigation in larger series might clarify the role of this miRNA in migraine.

To our knowledge, this was the first study which sought an association between the expression levels of a wide range of miRNAs and the degree of response to a migraine-specific preventive treatment. The seemingly erratic expression of miRNAs in response to treatment in women with CM could depend on the presence in some patients of medication overuse or other factors associated with central sensitization [36] which could alter the biology of migraine. EM is less associated with central sensitization phenomena and therefore might have a more predictable course and fewer challenges in the response to treatment. By analyzing a considerable panel of miRNAs subjected to the analysis, several microRNA, related to the disease burden and/or to treatment administration, emerged as interesting putative biomarkers, offering novel data to be further explored. Additionally, our data confirmed on circulating miRNAs the same findings that were reported in peripheral blood cells in patients with CM [16]. Despite these strengths, our study is not without limitations. Firstly, we only assessed women treated with erenumab, the first CGRP-mAbs to be marketed and the only one which was available at the time the protocol was conceived. Other CGRP-mAbs or gepants can induce different changes in miRNA expression compared with erenumab. The sample size was relatively modest, which curtailed the utility of subgroup analyses. Larger sample sizes could have increased the number of miRNAs with significant expression changes in the different analyses. Furthermore, the dropout of some patients, particularly in the CM group, further impacted the overall study sample size. In addition, our study was confined to women aged 25–50 years, which ensured better uniformity in miRNA expression, while on the other hand limiting the generalizability of our findings to the entire spectrum of patients with migraine. Concomitant medications were unchanged during the study to minimize confounding; however, we cannot exclude the possibility of a subtle effect of confounders. Our study did not include a group of controls without migraine which could corroborate the changes in miRNA expression levels found in participants with migraine. Another limitation could be attributed to the relatively brief duration of treatment; additional studies taking into consideration longer therapy administration periods of time might reveal additional interesting miRNAs. Furthermore, the possibility to adopt recently refined high-throughput next-generation sequencing technologies, such as miRNome sequencing, could go deeper into the analysis, possibly leading to the identification of further miRNAs playing a role in the migraine and its therapeutic response. We excluded women with major comorbidities; however, unrecognized or untreated comorbidities might have modulated miRNAs independently from migraine and its treatment. Lastly, we included women with medication overuse, which might have been associated with epigenetic alterations independent from those of migraine.

## Conclusions

In conclusion, our findings suggest that miRNA expression undergoes changes following a brief course of a CGRP-targeting treatment. Two miRNAs—*hsa-miR-34a-5p* and *hsa-miR-382-5p* – were able to distinguish women with CM from those with EM and showed changes from baseline to post-treatment only in women with CM. H*sa-miR-143-3p* emerged as a putative biomarker of response to migraine prevention with a CGRP-targeting treatment in women with EM. Subsequent confirmatory investigations on large patients’ series are needed to confirm the potential role of the identified miRNAs as biomarkers for migraine types and their treatment. In addition, a challenge in future research will be to ascribe specific functions to the modulated miRNAs, thereby uncovering novel pathological pathways and potential pharmacological targets in migraine.

### Supplementary Information

Supplementary Material 1: Supplementary Table 1. List of microRNAs analyzed in the present study (TaqMan Advanced miRNA Human Serum/Plasma Cards, Thermo Fisher).

Supplementary Material 2: Supplementary Table 2. Efficacy data of erenumab in women with episodic migraine and chronic migraine in the present study.

## Data Availability

Data for the present study are available from the Corresponding Author upon reasonable request.
