# Supplementary material for: MicroRNA profiling in women with migraine: effects of CGRP-targeting treatment
Source: J Headache Pain. 2024 May 16;25(1):80. doi: 10.1186/s10194-024-01787-2 (PMC11100066; doi:10.1186/s10194-024-01787-2)
Supplement: Supplementary file 1 — Supplementary Material 1: Supplementary Table 1. List of microRNAs analyzed in the present study (TaqMan Advanced miRNA Human Serum/Plasma Cards, Thermo Fisher). [file 10194_2024_1787_MOESM1_ESM.docx]

**Supplementary Table 1**. List of microRNAs analyzed in the present study (TaqMan Advanced miRNA Human Serum/Plasma Cards, Thermo Fisher)

| **ASSAY_ID** | **TARGET_NAME** | **MIRNA_SEQ** |
| --- | --- | --- |
| 478575_mir | hsa-let-7a-5p | UGAGGUAGUAGGUUGUAUAGUU |
| 478221_mir | hsa-let-7b-3p | CUAUACAACCUACUGCCUUCCC |
| 478576_mir | hsa-let-7b-5p | UGAGGUAGUAGGUUGUGUGGUU |
| 478577_mir | hsa-let-7c-5p | UGAGGUAGUAGGUUGUAUGGUU |
| 477848_mir | hsa-let-7d-3p | CUAUACGACCUGCUGCCUUUCU |
| 478439_mir | hsa-let-7d-5p | AGAGGUAGUAGGUUGCAUAGUU |
| 478579_mir | hsa-let-7e-5p | UGAGGUAGGAGGUUGUAUAGUU |
| 478578_mir | hsa-let-7f-5p | UGAGGUAGUAGAUUGUAUAGUU |
| 478580_mir | hsa-let-7g-5p | UGAGGUAGUAGUUUGUACAGUU |
| 477862_mir | hsa-let-7i-3p | CUGCGCAAGCUACUGCCUUGCU |
| 477860_mir | hsa-miR-16-5p | UAGCAGCACGUAAAUAUUGGCG |
| 478375_mir | hsa-let-7i-5p | UGAGGUAGUAGUUUGUGCUGUU |
| 477820_mir | hsa-miR-1-3p | UGGAAUGUAAAGAAGUAUGUAU |
| 477863_mir | hsa-miR-101-3p | UACAGUACUGUGAUAACUGAA |
| 478253_mir | hsa-miR-103a-3p | AGCAGCAUUGUACAGGGCUAUGA |
| 478225_mir | hsa-miR-106a-5p | AAAAGUGCUUACAGUGCAGGUAG |
| 477866_mir | hsa-miR-106b-3p | CCGCACUGUGGGUACUUGCUGC |
| 478412_mir | hsa-miR-106b-5p | UAAAGUGCUGACAGUGCAGAU |
| 478254_mir | hsa-miR-107 | AGCAGCAUUGUACAGGGCUAUCA |
| 479241_mir | hsa-miR-10a-5p | UACCCUGUAGAUCCGAAUUUGUG |
| 478494_mir | hsa-miR-10b-5p | UACCCUGUAGAACCGAAUUUGUG |
| 477855_mir | hsa-miR-122-5p | UGGAGUGUGACAAUGGUGUUUG |
| 477884_mir | hsa-miR-125a-5p | UCCCUGAGACCCUUUAACCUGUGA |
| 477885_mir | hsa-miR-125b-5p | UCCCUGAGACCCUAACUUGUGA |
| 477887_mir | hsa-miR-126-3p | UCGUACCGUGAGUAAUAAUGCG |
| 477889_mir | hsa-miR-127-3p | UCGGAUCCGUCUGAGCUUGGCU |
| 477892_mir | hsa-miR-128-3p | UCACAGUGAACCGGUCUCUUU |
| 477851_mir | hsa-miR-130a-3p | CAGUGCAAUGUUAAAAGGGCAU |
| 477840_mir | hsa-miR-130b-3p | CAGUGCAAUGAUGAAAGGGCAU |
| 477900_mir | hsa-miR-132-3p | UAACAGUCUACAGCCAUGGUCG |
| 478511_mir | hsa-miR-133a-3p | UUUGGUCCCCUUCAACCAGCUG |
| 480871_mir | hsa-miR-133b | UUUGGUCCCCUUCAACCAGCUA |
| 478307_mir | hsa-miR-136-5p | ACUCCAUUUGUUUUGAUGAUGGA |
| 478312_mir | hsa-miR-139-5p | UCUACAGUGCACGUGUCUCCAGU |
| 477908_mir | hsa-miR-140-3p | UACCACAGGGUAGAACCACGG |
| 477909_mir | hsa-miR-140-5p | CAGUGGUUUUACCCUAUGGUAG |
| 478501_mir | hsa-miR-141-3p | UAACACUGUCUGGUAAAGAUGG |
| 477910_mir | hsa-miR-142-3p | UGUAGUGUUUCCUACUUUAUGGA |
| 477911_mir | hsa-miR-142-5p | CAUAAAGUAGAAAGCACUACU |
| 477912_mir | hsa-miR-143-3p | UGAGAUGAAGCACUGUAGCUC |
| 477913_mir | hsa-miR-144-3p | UACAGUAUAGAUGAUGUACU |
| 477914_mir | hsa-miR-144-5p | GGAUAUCAUCAUAUACUGUAAG |
| 477916_mir | hsa-miR-145-5p | GUCCAGUUUUCCCAGGAAUCCCU |
| 478399_mir | hsa-miR-146a-5p | UGAGAACUGAAUUCCAUGGGUU |
| 478513_mir | hsa-miR-146b-5p | UGAGAACUGAAUUCCAUAGGCU |
| 477814_mir | hsa-miR-148a-3p | UCAGUGCACUACAGAACUUUGU |
| 477824_mir | hsa-miR-148b-3p | UCAGUGCAUCACAGAACUUUGU |
| 477918_mir | hsa-miR-150-5p | UCUCCCAACCCUUGUACCAGUG |
| 477919_mir | hsa-miR-151a-3p | CUAGACUGAAGCUCCUUGAGG |
| 478505_mir | hsa-miR-151a-5p | UCGAGGAGCUCACAGUCUAGU |
| 477921_mir | hsa-miR-152-3p | UCAGUGCAUGACAGAACUUGG |
| 477925_mir | hsa-miR-154-5p | UAGGUUAUCCGUGUUGCCUUCG |
| 477927_mir | hsa-miR-155-5p | UUAAUGCUAAUCGUGAUAGGGGU |
| 477858_mir | hsa-miR-15a-5p | UAGCAGCACAUAAUGGUUUGUG |
| 477929_mir | hsa-miR-15b-3p | CGAAUCAUUAUUUGCUGCUCUA |
| 478313_mir | hsa-miR-15b-5p | UAGCAGCACAUCAUGGUUUACA |
| 477931_mir | hsa-miR-16-2-3p | CCAAUAUUACUGUGCUGCUUUA |
| 478447_mir | hsa-miR-17-5p | CAAAGUGCUUACAGUGCAGGUAG |
| 478411_mir | ath-miR159a | UUUGGAUUGAAGGGAGCUCUA |
| 477857_mir | hsa-miR-181a-5p | AACAUUCAACGCUGUCGGUGAGU |
| 477935_mir | hsa-miR-182-5p | UUUGGCAAUGGUAGAACUCACACU |
| 477939_mir | hsa-miR-185-5p | UGGAGAGAAAGGCAGUUCCUGA |
| 477940_mir | hsa-miR-186-5p | CAAAGAAUUCUCCUUUUGGGCU |
| 477944_mir | hsa-miR-18a-3p | ACUGCCCUAAGUGCUCCUUCUGG |
| 478551_mir | hsa-miR-18a-5p | UAAGGUGCAUCUAGUGCAGAUAG |
| 478584_mir | hsa-miR-18b-5p | UAAGGUGCAUCUAGUGCAGUUAG |
| 478358_mir | hsa-miR-190a-5p | UGAUAUGUUUGAUAUAUUAGGU |
| 477952_mir | hsa-miR-191-5p | CAACGGAAUCCCAAAAGCAGCUG |
| 478262_mir | hsa-miR-192-5p | CUGACCUAUGAAUUGACAGCC |
| 478314_mir | hsa-miR-193b-3p | AACUGGCCCUCAAAGUCCCGCU |
| 477956_mir | hsa-miR-194-5p | UGUAACAGCAACUCCAUGUGGA |
| 477957_mir | hsa-miR-195-5p | UAGCAGCACAGAAAUAUUGGC |
| 477959_mir | hsa-miR-197-3p | UUCACCACCUUCUCCACCCAGC |
| 477961_mir | hsa-miR-199a-3p_hsa-miR-199b-3p | ACAGUAGUCUGCACAUUGGUUA |
| 478231_mir | hsa-miR-199a-5p | CCCAGUGUUCAGACUACCUGUUC |
| 479228_mir | hsa-miR-19a-3p | UGUGCAAAUCUAUGCAAAACUGA |
| 478264_mir | hsa-miR-19b-3p | UGUGCAAAUCCAUGCAAAACUGA |
| 478490_mir | hsa-miR-200a-3p | UAACACUGUCUGGUAACGAUGU |
| 478351_mir | hsa-miR-200c-3p | UAAUACUGCCGGGUAAUGAUGGA |
| 478491_mir | hsa-miR-204-5p | UUCCCUUUGUCAUCCUAUGCCU |
| 477967_mir | hsa-miR-205-5p | UCCUUCAUUCCACCGGAGUCUG |
| 477819_mir | hsa-miR-208a-3p | AUAAGACGAGCAAAAAGCUUGU |
| 478317_mir | hsa-miR-20a-3p | ACUGCAUUAUGAGCACUUAAAG |
| 478586_mir | hsa-miR-20a-5p | UAAAGUGCUUAUAGUGCAGGUAG |
| 477804_mir | hsa-miR-20b-5p | CAAAGUGCUCAUAGUGCAGGUAG |
| 477973_mir | hsa-miR-21-3p | CAACACCAGUCGAUGGGCUGU |
| 477975_mir | hsa-miR-21-5p | UAGCUUAUCAGACUGAUGUUGA |
| 477970_mir | hsa-miR-210-3p | CUGUGCGUGUGACAGCGGCUGA |
| 477971_mir | hsa-miR-2110 | UUGGGGAAACGGCCGCUGAGUG |
| 478516_mir | hsa-miR-215-5p | AUGACCUAUGAAUUGACAGAC |
| 477985_mir | hsa-miR-22-3p | AAGCUGCCAGUUGAAGAACUGU |
| 477987_mir | hsa-miR-22-5p | AGUUCUUCAGUGGCAAGCUUUA |
| 477981_mir | hsa-miR-221-3p | AGCUACAUUGUCUGCUGGGUUUC |
| 477982_mir | hsa-miR-222-3p | AGCUACAUCUGGCUACUGGGU |
| 477983_mir | hsa-miR-223-3p | UGUCAGUUUGUCAAAUACCCCA |
| 477984_mir | hsa-miR-223-5p | CGUGUAUUUGACAAGCUGAGUU |
| 477986_mir | hsa-miR-224-5p | CAAGUCACUAGUGGUUCCGUU |
| 478532_mir | hsa-miR-23a-3p | AUCACAUUGCCAGGGAUUUCC |
| 478602_mir | hsa-miR-23b-3p | AUCACAUUGCCAGGGAUUACC |
| 477992_mir | hsa-miR-24-3p | UGGCUCAGUUCAGCAGGAACAG |
| 477994_mir | hsa-miR-25-3p | CAUUGCACUUGUCUCGGUCUGA |
| 477995_mir | hsa-miR-26a-5p | UUCAAGUAAUCCAGGAUAGGCU |
| 478418_mir | hsa-miR-26b-5p | UUCAAGUAAUUCAGGAUAGGU |
| 478384_mir | hsa-miR-27a-3p | UUCACAGUGGCUAAGUUCCGC |
| 478270_mir | hsa-miR-27b-3p | UUCACAGUGGCUAAGUUCUGC |
| 477999_mir | hsa-miR-28-3p | CACUAGAUUGUGAGCUCCUGGA |
| 478293_mir | cel-miR-39-3p | UCACCGGGUGUAAAUCAGCUUG |
| 478000_mir | hsa-miR-28-5p | AAGGAGCUCACAGUCUAUUGAG |
| 477836_mir | hsa-miR-296-5p | AGGGCCCCCCCUCAAUCCUGU |
| 478587_mir | hsa-miR-29a-3p | UAGCACCAUCUGAAAUCGGUUA |
| 478002_mir | hsa-miR-29a-5p | ACUGAUUUCUUUUGGUGUUCAG |
| 478003_mir | hsa-miR-29b-2-5p | CUGGUUUCACAUGGUGGCUUAG |
| 478369_mir | hsa-miR-29b-3p | UAGCACCAUUUGAAAUCAGUGUU |
| 479229_mir | hsa-miR-29c-3p | UAGCACCAUUUGAAAUCGGUUA |
| 477815_mir | hsa-miR-301a-3p | CAGUGCAAUAGUAUUGUCAAAGC |
| 477825_mir | hsa-miR-301b-3p | CAGUGCAAUGAUAUUGUCAAAGC |
| 479448_mir | hsa-miR-30a-5p | UGUAAACAUCCUCGACUGGAAG |
| 478007_mir | hsa-miR-30b-5p | UGUAAACAUCCUACACUCAGCU |
| 478008_mir | hsa-miR-30c-5p | UGUAAACAUCCUACACUCUCAGC |
| 478606_mir | hsa-miR-30d-5p | UGUAAACAUCCCCGACUGGAAG |
| 478388_mir | hsa-miR-30e-3p | CUUUCAGUCGGAUGUUUACAGC |
| 479235_mir | hsa-miR-30e-5p | UGUAAACAUCCUUGACUGGAAG |
| 478015_mir | hsa-miR-31-5p | AGGCAAGAUGCUGGCAUAGCU |
| 478026_mir | hsa-miR-32-5p | UAUUGCACAUUACUAAGUUGCA |
| 478594_mir | hsa-miR-320a | AAAAGCUGGGUUGAGAGGGCGA |
| 478588_mir | hsa-miR-320b | AAAAGCUGGGUUGAGAGGGCAA |
| 478022_mir | hsa-miR-320e | AAAGCUGGGUUGAGAAGG |
| 478023_mir | hsa-miR-324-3p | ACUGCCCCAGGUGCUGCUGG |
| 478024_mir | hsa-miR-324-5p | CGCAUCCCCUAGGGCAUUGGUGU |
| 478027_mir | hsa-miR-326 | CCUCUGGGCCCUUCCUCCAG |
| 478028_mir | hsa-miR-328-3p | CUGGCCCUCUCUGCCCUUCCGU |
| 478323_mir | hsa-miR-331-3p | GCCCCUGGGCCUAUCCUAGAA |
| 478324_mir | hsa-miR-335-5p | UCAAGAGCAAUAACGAAAAAUGU |
| 478037_mir | hsa-miR-338-3p | UCCAGCAUCAGUGAUUUUGUUG |
| 478325_mir | hsa-miR-339-3p | UGAGCGCCUCGACGACAGAGCCG |
| 478040_mir | hsa-miR-339-5p | UCCCUGUCCUCCAGGAGCUCACG |
| 478347_mir | hsa-miR-33a-5p | GUGCAUUGUAGUUGCAUUGCA |
| 478043_mir | hsa-miR-342-3p | UCUCACACAGAAAUCGCACCCGU |
| 478046_mir | hsa-miR-346 | UGUCUGCCCGCAUGCCUGCCUCU |
| 478048_mir | hsa-miR-34a-5p | UGGCAGUGUCUUAGCUGGUUGU |
| 478055_mir | hsa-miR-361-3p | UCCCCCAGGUGUGAUUCUGAUUU |
| 478060_mir | hsa-miR-363-3p | AAUUGCACGGUAUCCAUCUGUA |
| 478065_mir | hsa-miR-365a-3p_hsa-miR-365b-3p | UAAUGCCCCUAAAAAUCCUUAU |
| 478363_mir | hsa-miR-373-3p | GAAGUGCUUCGAUUUUGGGGUGU |
| 478073_mir | hsa-miR-373-5p | ACUCAAAAUGGGGGCGCUUUCC |
| 478238_mir | hsa-miR-374a-5p | UUAUAAUACAACCUGAUAAGUG |
| 478389_mir | hsa-miR-374b-5p | AUAUAAUACAACCUGCUAAGUG |
| 478074_mir | hsa-miR-375 | UUUGUUCGUUCGGCUCGCGUGA |
| 478240_mir | hsa-miR-376a-3p | AUCAUAGAGGAAAAUCCACGU |
| 478349_mir | hsa-miR-378a-3p | ACUGGACUUGGAGUCAGAAGGC |
| 478078_mir | hsa-miR-382-5p | GAAGUUGUUCGUGGUGGAUUCG |
| 478084_mir | hsa-miR-409-3p | GAAUGUUGCUCGGUGAACCCCU |
| 478088_mir | hsa-miR-421 | AUCAACAGACAUUAAUUGGGCGC |
| 478327_mir | hsa-miR-423-3p | AGCUCGGUCUGAGGCCCCUCAGU |
| 478090_mir | hsa-miR-423-5p | UGAGGGGCAGAGAGCGAGACUUU |
| 478092_mir | hsa-miR-424-5p | CAGCAGCAAUUCAUGUUUUGAA |
| 478093_mir | hsa-miR-425-3p | AUCGGGAAUGUCGUGUCCGCCC |
| 478094_mir | hsa-miR-425-5p | AAUGACACGAUCACUCCCGUUGA |
| 478107_mir | hsa-miR-451a | AAACCGUUACCAUUACUGAGUU |
| 478122_mir | hsa-miR-483-3p | UCACUCCUCUCCUCCCGUCUU |
| 478432_mir | hsa-miR-483-5p | AAGACGGGAGGAAAGAAGGGAG |
| 478308_mir | hsa-miR-484 | UCAGGCUCAGUCCCCUCCCGAU |
| 478125_mir | hsa-miR-485-3p | GUCAUACACGGCUCUCCUCUCU |
| 478128_mir | hsa-miR-486-5p | UCCUGUACUGAGCUGCCCCGAG |
| 478136_mir | hsa-miR-495-3p | AAACAAACAUGGUGCACUUCUU |
| 478138_mir | hsa-miR-497-5p | CAGCAGCACACUGUGGUUUGU |
| 478309_mir | hsa-miR-500a-5p | UAAUCCUUGCUACCUGGGUGAGA |
| 478350_mir | hsa-miR-501-3p | AAUGCACCCGGGCAAGGAUUCU |
| 478348_mir | hsa-miR-502-3p | AAUGCACCUGGGCAAGGAUUCA |
| 478145_mir | hsa-miR-505-3p | CGUCAACACUUGCUGGUUUCCU |
| 478336_mir | hsa-miR-532-3p | CCUCCCACACCCAAGGCUUGCA |
| 478151_mir | hsa-miR-532-5p | CAUGCCUUGAGUGUAGGACCGU |
| 478155_mir | hsa-miR-543 | AAACAUUCGCGGUGCACUUCUU |
| 478158_mir | hsa-miR-551a | GCGACCCACUCUUGGUUUCCA |
| 478159_mir | hsa-miR-551b-3p | GCGACCCAUACUUGGUUUCAG |
| 478163_mir | hsa-miR-574-3p | CACGCUCAUGCACACACCCACA |
| 478167_mir | hsa-miR-584-5p | UUAUGGUUUGCCUGGGACUGAG |
| 478367_mir | hsa-miR-590-5p | GAGCUUAUUCAUAAAAGUGCAG |
| 478174_mir | hsa-miR-605-5p | UAAAUCCCAUGGUGCCUUCUCCU |
| 478183_mir | hsa-miR-629-5p | UGGGUUUACGUUGGGAGAACU |
| 478189_mir | hsa-miR-652-3p | AAUGGCGCCACUAGGGUUGUG |
| 478192_mir | hsa-miR-660-5p | UACCCAUUGCAUAUCGGAGUUG |
| 478342_mir | hsa-miR-766-3p | ACUCCAGCCCCACAGCCUCAGC |
| 478207_mir | hsa-miR-885-5p | UCCAUUACACUACCCUGCCUCU |
| 477827_mir | hsa-miR-92a-3p | UAUUGCACUUGUCCCGGCCUGU |
| 477823_mir | hsa-miR-92b-3p | UAUUGCACUCGUCCCGGCCUCC |
| 478209_mir | hsa-miR-93-3p | ACUGCUGAGCUAGCACUUCCCG |
| 478210_mir | hsa-miR-93-5p | CAAAGUGCUGUUCGUGCAGGUAG |
| 478213_mir | hsa-miR-95-3p | UUCAACGGGUAUUUAUUGAGCA |
| 478519_mir | hsa-miR-99a-5p | AACCCGUAGAUCCGAUCUUGUG |
| 478343_mir | hsa-miR-99b-5p | CACCCGUAGAACCGACCUUGCG |
