# Supplementary material for: MicroRNA profiling in women with migraine: effects of CGRP-targeting treatment
Source: J Headache Pain. 2024 May 16;25(1):80. doi: 10.1186/s10194-024-01787-2 (PMC11100066; doi:10.1186/s10194-024-01787-2)
Supplement: Supplementary file 2 — Supplementary Material 2: Supplementary Table 2. Efficacy data of erenumab in women with episodic migraine and chronic migraine in the present study. [file 10194_2024_1787_MOESM2_ESM.docx]

**Supplementary Table 2**. Efficacy data of erenumab in women with episodic migraine and chronic migraine in the present study.

| **Variable** | **Baseline** | **Weeks 1-4** | **Weeks 5-8** | **Weeks 9-12** |
| --- | --- | --- | --- | --- |
| *Episodic migraine (n=19)* |  |  |  |  |
| Monthly headache days | 10.3±5.0 | 6.7±4.5 (p=0.025) | 5.8±4.1 (p=0.005) | 6.1±5.7 (p=0.021) |
| Monthly migraine days | 9.0±2.8 | 4.7±3.7 (p<0.001) | 4.5±3.5 (p<0.001) | 4.8±4.1 (p=0.001) |
| Monthly days of acute medication use | 8.7±3.0 | 4.3±3.8 (p<0.001) | 4.2±3.0 (p<0.001) | 4.7±5.4 (p=0.009) |
| Monthly acute medication intakes | 10.2±4.5 | 5.7±5.0 (p=0.006) | 5.4±4.0 (p=0.001) | 7.8±12.8 (p=0.449) |
| MIDAS score | 78.9±48.6 | - | - | 24.8±26.7 (p<0.001) |
| HIT-6 score | 68.7±4.0 | - | - | 61.6±7.7 (p=0.002) |
| BDI score | 11.3±7.2 | - | - | 7.4±7.6 (p=0.001) |
| ASC-12 score | 6.4±2.6 | - | - | 5.2±3.2 (p=0.213) |
| PSQI score | 8.0±4.0 | - | - | 7.3±3.7 (p=0.579) |
| *Episodic migraine (n=19)* |  |  |  |  |
| Monthly headache days | 19.9±6.2 | 13.4±9.2 (p=0.022) | 12.1±9.6 (p=0.009) | 10.5±8.8 (p=0.001) |
| Monthly migraine days | 16.8±7.6 | 10.5±8.9 (p=0.034) | 9.8±9.6 (p=0.025) | 8.5±8.9 (p=0.006) |
| Monthly days of acute medication use | 13.5±7.3 | 7.9±6.0 (p=0.020) | 6.4±6.2 (p=0.005) | 6.4±6.1 (p=0.004) |
| Monthly acute medication intakes | 18.5±13.2 | 9.6±7.5 (p=0.023) | 7.5±7.5 (p=0.006) | 7.9±7.6 (p=0.008) |
| MIDAS score | 113.2±77.4 | - | - | 57.9±81.1 (p=0.050) |
| HIT-6 score | 68.1±3.2 | - | - | 60.9±7.1 (p=0.001) |
| BDI score | 15.2±10.6 | - | - | 10.9±8.8 (p=0.208) |
| ASC-12 score | 9.2±4.2 | - | - | 6.4±4.8 (p=0.080) |
| PSQI score | 9.6±4.0 | - | - | 7.9±3.8 (p=0.213) |
